# Pattern of resistance on first-line EGFR-directed therapy in EGFR-positive metastatic NSCLC

**DOI:** 10.3332/ecancer.2026.2086

**Published:** 2026-03-09

**Authors:** Sree Siva Kumar Raja Addagalla, Vanita Noronha, Nandini Menon, Minit Shah, Amit Joshi, Anokhi Shah, Kumar Prabhash

**Affiliations:** Department of Medical Oncology, Tata Memorial Centre, Homi Bhabha National Institute (HBNI), Mumbai 400094, India; https://orcid.org/0009-0009-0042-8609; https://orcid.org/0000-0002-4214-5799; https://orcid.org/0009-0004-3769-3194; https://orcid.org/0000-0001-8858-5004

**Keywords:** resistance, EGFR-TKIs, NSCLC, T790M, chemotherapy

## Abstract

**Background and purpose:**

Resistance to epithelial growth factor receptor gene (EGFR)-tyrosine kinase inhibitors, both intrinsic and acquired, presents a major challenge in EGFR mutant non-small cell lung carcinoma (NSCLC), with the T790M mutation being the most common acquired resistance mechanism. Data on resistance patterns in the Indian population remains limited.

**Materials and methods:**

This *post hoc* analysis of a Phase III trial conducted at Tata Memorial Centre (TMC) in Mumbai, India, included 350 patients with advanced EGFR-mutant NSCLC. Patients were randomised to receive either gefitinib alone (*n* = 176) or gefitinib with chemotherapy (pemetrexed and carboplatin, *n* = 174). The primary objective was to identify resistance mechanisms following progression. Secondary objectives included comparing resistance patterns between the two arms and assessing progression rates and outcomes. At progression, histological and molecular evaluation was done with reverse transcription polymerase chain reaction (RT-PCR), anaplastic lymphoma kinase – immunohistochemistry (ALK IHC) and/or next generation sequencing (NGS) at physician discretion.

**Results:**

Of the 275 (78.6%) patients experiencing progressive disease (PD), a total of 206 patients were available for the final analysis after excluding patients without histological/molecular analysis.

Histological transformation to small cell lung carcinoma (SCLC) occurred in 12 patients (6%), with various EGFR mutation statuses identified. T790M mutations were observed in 44 (37%) of 119 patients in the gefitinib arm and 17 (19%) of 87 patients in the gefitinib plus chemotherapy arm (*p* = 0.008). A new sensitising mutation was found in 6 (5%) in the gefitinib arm and in 1 (1.1%) in the gefitinib plus chemotherapy arm (*p* – 0.12). Loss of prior sensitising mutation was found in 21 (17.6%) in the gefitinib arm and in 28 (32%) in the gefitinib plus chemotherapy arm (*p* – 0.015).

Patients with T790M mutations had a progression-free survival 2 of 22.7 months (95% CI: 19.4–27.4 months), compared to 19.2 months (95% CI: 17.5–22.9 months) in those without T790M mutations (*p* = 0.95). Overall survival was 27.9 months (95% CI: 24.6–34.6 months) in the T790M group compared to 26.5 months (95% CI: 23.2–30.1 months) in the non-T790M group (*p* = 0.75).

**Interpretation:**

Emergence of T790M was lower than reported in previous studies, likely due to the addition of chemotherapy to gefitinib. T790M mutations were more prevalent in the gefitinib-alone arm. Histological transformation and loss of sensitising mutations highlight the importance of repeat biopsy and molecular testing to guide subsequent treatment decisions.

## Introduction

The identification of driver mutations like epithelial growth factor receptor gene *(EGFR), ALK* and subsequent development of effective tyrosine kinase inhibitors (TKIs) against them has transformed therapy in metastatic non-small cell lung carcinoma (NSCLC) [1]. The incidence of *EGFR*-mutated NSCLC was found to be about 45.8% in the Indian population [2]. EGFR is a tyrosine kinase receptor which binds to its ligand epidermal growth factor, leading to receptor dimerisation, auto-phosphorylation at the tyrosine kinase residues, activating cellular signaling pathways such as the phosphoinositide 3-kinase-protein kinase B pathway, the signal transducer and activator of transcription pathway and the mitogen-activated protein kinase pathway, resulting in increased cell proliferation, migration and survival [3–5]. *EGFR*-activating gene mutations cause constitutive EGFR activation, which leads to cell proliferation. The most common mutations occur in exons 19 and 21 accounting for approximately 45% and 40% of all *EGFR* mutations, respectively, and are associated with good responses to EGFR-targeted small molecule inhibitor therapies [5]. Less common mutations include G719X in exon 18, L861Q in exon 21, and insertions in exon 20 (Ex20ins) are each estimated to constitute about 2% of *EGFR* mutations [6–8]. 

Initial studies in *EGFR* mutant NSCLC have shown better outcomes with the first and second generation TKIs compared to chemotherapy [9–15]. In the Indian setting, our team at Tata Memorial Hospital, (Mumbai, India) showed that combination of chemotherapy with the first generation TKI (gefitinib) improved survival [16]. Subsequently, the third generation EGFR-TKI, osimertinib, was introduced, which was active against the T790M resistance mutation [17]. It was also shown to be efficacious in the first line setting, with a superior overall survival (OS) and progression free survival (PFS) as compared to first generation EGFR TKIs as demonstrated in the FLAURA trial [17, 18]. In the FLAURA 2 trial, osimertinib plus chemotherapy (pemetrexed with cisplatin or carboplatin) was compared against osimertinib and this showed a PFS benefit with chemotherapy (25.5 versus 16.7 months , HR 0.62; *p* < 0.01) and at 2 years, the OS was not reached versus 36.7 months (HR, 0.75; 95% CI, 0.57–0.97; *p* = 0.0280 with an overall maturity of 41%) with increased hematological toxicity [19]. In the MARIPOSA trial, amivantamab with lazertinib improved median PFS (23.7 versus 16.6 months, HR 0.7 *p* < 0.001) and improved interim OS (HR 0.8) when compared to osimertinib [20]. Despite superior outcomes of these newer agents, high cost and less availability limit their use in routine clinical practice. Osimertinib costs about 3.2 lakhs INR per month. Amivantamab costs about 10 lakhs INR in the first month and 5 lakhs per month in the subsequent months and lazertinib is currently not marketed in India. This situation led to the use of early generation TKIs alone or in combination with chemotherapy. But after an initial response, they invariably lead to resistance.

Resistance to EGFR TKIs can be classified into two major categories: intrinsic and acquired. Intrinsic resistance accounts for 20%–30% of cases and is related to poor initial response to TKIs. It can be attributed to pre-existing de novo EGFR-dependent or independent mechanisms, whereas the acquired resistance can arise under therapeutic selective pressure, as a result of either the expansion of pre-existing sub-clonal populations or the evolution of drug-tolerant cells [20]. Different mechanisms of acquired resistance to first-generation EGFR-TKIs have been reported [21]. EGFR T790M mutation is the most common mechanism occurring in 50%–60% of cases [21–24]. Although, a number of studies have been done to elucidate the patterns of resistance in the patients who have progressed on EGFR-TKIs, such data in Indian population is sparse and remains an unmet need.

## Materials and methods

### General study details

This was a *post hoc* analysis of the randomised, open-label, phase III study that compared gefitinib with gefitinib plus chemotherapy in patients with advanced NSCLC with activating EGFR mutations in the first-line setting. The methodology has been published in detail already [16]. The trial was conducted at TMC, an academic tertiary oncology hospital in Mumbai, India. A total of 350 patients were enrolled in the study. The study was approved by the Institutional Ethics Committee of TMC and monitored by the Data Safety Monitoring Subcommittee. All patients provided written informed consent. The trial was conducted according to the principles laid down by the International Conference on Harmonisation Good Clinical Practice guidelines, the Declaration of Helsinki, and Schedule Y (Drugs and Cosmetic Act, 1940) and the guidelines established by the Indian Council of Medical Research. The trial was registered at Clinical Trials Registry–India (identifier: CTRI/ 2016/08/007149).

### Participants

The patients were randomised to receive either Gefitinib alone or Gefitinib along with chemotherapy. Patients who had progressed underwent re-biopsy, depending on feasibility.

### Study aim/objective

Our primary objective was to describe the mechanisms of resistance in patients who progressed on either gefitinib alone or in combination with chemotherapy. The secondary objectives included the differences in the resistance patterns between patients who progressed on gefitinib alone versus those who progressed on gefitinib along with chemotherapy, the patterns of progression and progression rates among various *EGFR* mutations and factors affecting outcomes.

### Study methodology

At progression (clinical or radiological), all the patients were evaluated for the feasibility of re-biopsy. In patients in whom biopsy was not possible, if any fluid collection was present – cytology and cell block were obtained. All the patients were evaluated for the feasibility of liquid biopsy on blood and this was done whenever possible. We excluded the patients in whom neither tissue biopsy nor liquid biopsy were possible.

In the patients for whom either biopsy or cell block was available, histopathological evaluation and* EGFR* RT-PCR was done in all and ALK IHC and NGS was done in some of the patients when possible and affordable based on the physician discretion.

### Stastistics

Using descriptive statistical analysis, the patterns of progression, mechanisms of resistance and variations in their patterns between the gefitinib and gefitinib plus chemotherapy groups were computed. The difference in the emergence of T790M mutation between the two arms were compared using the Pearson chi-square test and a ‘*p*’ value of < 0.05 was considered as significant. Progression free survival 2 (PFS2) was calculated from the date of randomisation to either the date of second progression or date of last follow-up or date of death if there is no event of second progression using Kaplan Meir analysis with a ‘*p*’ value of < 0.05 being considered as significant. The event for PFS2 was taken as either second progression or death. OS was calculated from the date of randomisation to the date of death or date of last follow-up using Kaplan Meir analysis with a ‘*p*’ value of < 0.05 being considered as significant. Death was taken as the event for OS. Patients who were lost to follow-up were censored while calculating both OS and PFS2.

## Results

### Patient predisposition

350 patients who were enrolled between August 2016 and 2018 and 176 (50.3%) patients were assigned to gefitinib and 174 (49.7%) patients were assigned to Gefitinib along with pemetrexed and carboplatin. These patients were followed up over the period till May 2024.

### Baseline characteristics

The patients’ baseline characteristics with respect to demographic data and the molecular data are listed in Table 1 as follows.

**Figure 1.**
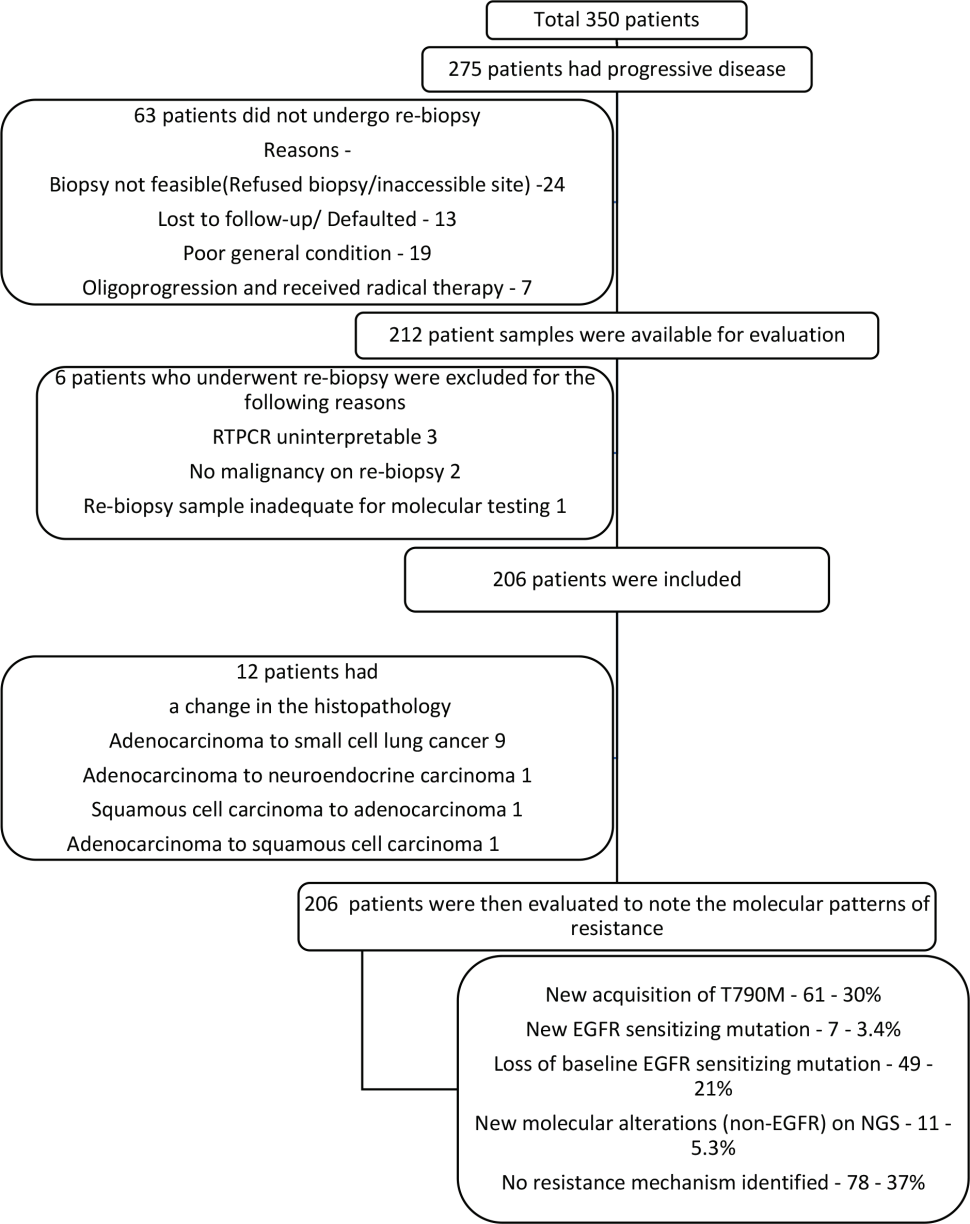
Consort diagram.

The outcomes were tabulated in the Supplementary Table 1, and the PFS among various molecular mutations were tabulated in Supplementary Table 2. The consort diagram was in Figure 1. Of the 275 patients who had PD (the sites of progression were tabulated in Table 2), 10 (3.7%) had subjective PD alone (imaging could not be performed for various reasons) and 265 (96.3%) were noted to have radiological PD. Of the 265, 110 (40%) had radiological PD alone and 155 (56.3%) had both radiological and subjective PD. The details of the sites of biopsy were tabulated in the Supplementary Table 4, while the complications were tabulated in Supplementary Table 5.

Tissue biopsies were obtained on 157 (57.1%) patients and a cell block in 14 (5.1%). Liquid biopsy was performed in 169 (61.5%) patients. The details of repeat biopsy given in the Table 3.

Of the nine patients who had histological transformation to small cell carcinoma, the results of molecular testing were 6 had Exon 19 retained, 1 had loss of exon 19, 1 had new mutation of S768I along with L858R retained, 1 had inadequate biopsy for molecular testing.

### Resistance patterns – gefitinib versus gefitinib plus chemotherapy

**Figure 2.**
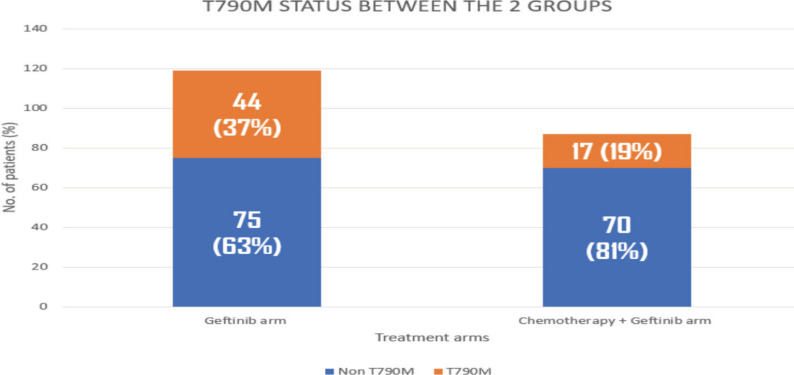
Histogram showing comparison of T790M in geftinib versus geftinib + chemotherapy arms.

**Figure 3.**
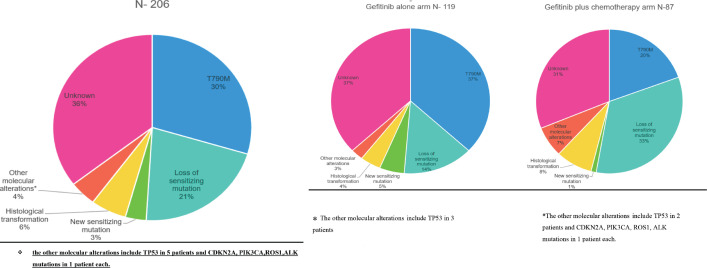
(a): Overall resistance mechanisms in the study population. (b): Resistance mechanisms in the gefitinib arm. (c): Resistance mechanisms in the gefitinib plus chemotherapy.

The molecular analysis of the patients at progression were tabulated in Supplementary Tables 6 and 7. Additional molecular analysis noted on NGS were tabulated in Supplementary Table 8. T790M has been noted in 44 (37%) out of 119 patients in the gefitinib arm, whereas it was noted in 17 (19%) out of 87 patients in the gefitinib plus chemotherapy arm (*p* – 0.008) Figure 2. Patients receiving chemotherapy plus gefitinib, SCLC transformation was found in 6 out of 87(7%), while it was found in 3 out of 119 patients (2%) in the gefitinib arm (*p* - 0.12). The comparison between the two groups is charted in Table 2, and the changes in the overall cohort and individual arms are depicted in Figure 3.

#### T790M status

**Figure S1.**
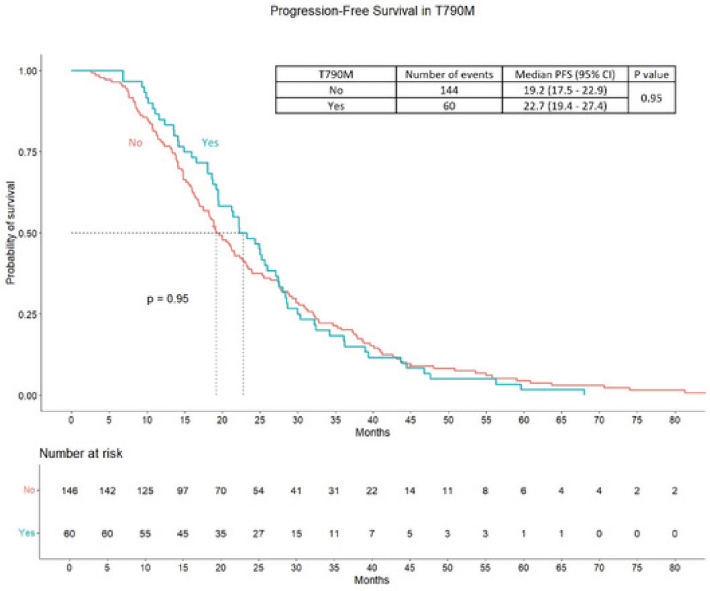
PFS in patients of T90M positive compared to other patients.

**Figure S2.**
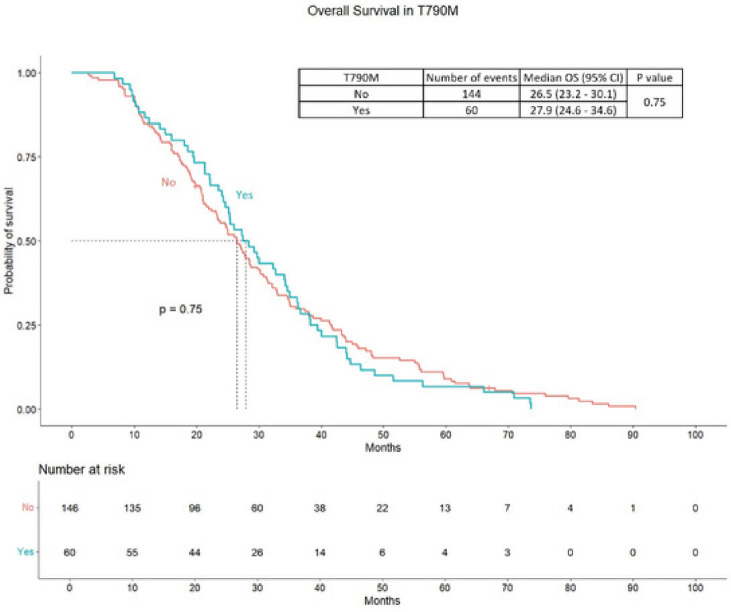
OS in patients of T90M positive compared to other patients.

PFS2 among the patients with acquired T790M was 22.7 months (95% CI – 19.4 – 27.4 months), while the PFS 2 in patients without T790M was 19.2 months (95% CI – 17.5 – 22.9 months) (*p* = 0.95). Majority of them received osimertinib at progression (48 out of 57(84.2%) patients with T790M). The OS was 27.9 months (95% CI – 24.6 – 34.6 months) in the patients with T790M compared to 26.5 months (95% CI – 23.2 – 30.1 months) in in patients without T790M (*p* – 0.75). The survival curves are provided in Supplementary Data Figures 1 and 2.

#### Site of progression

**Figure S3.**
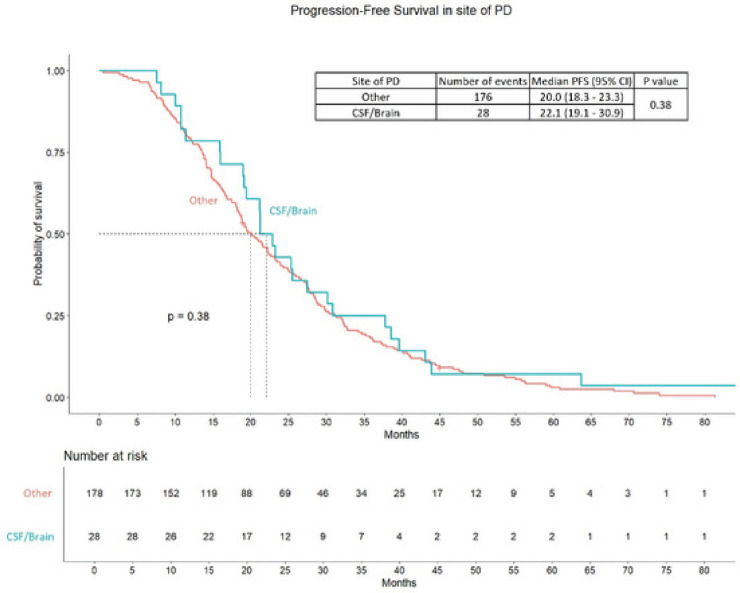
PFS difference between patients progressed in brain or CSF versus other sites of PD.

**Figure S4.**
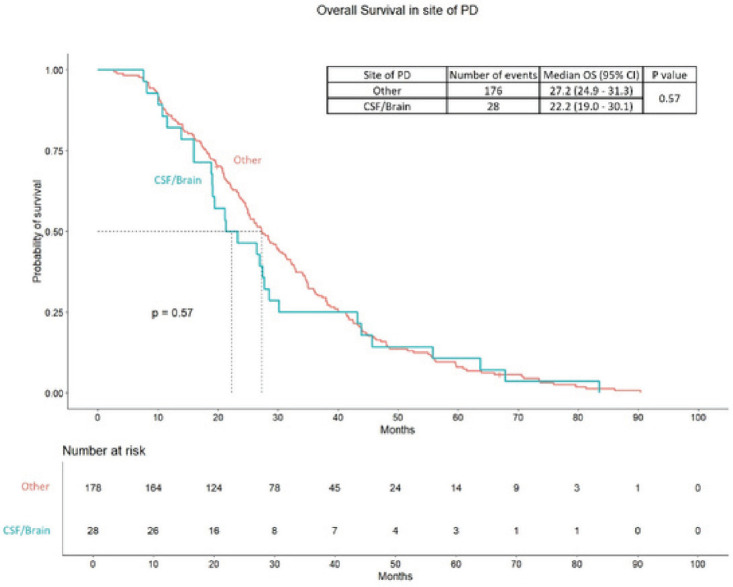
OS difference between patients progressed in the brain or CSF versus other sites of PD.

PFS2 among the patients with central nervous system (CNS) progression was 22.1 months (95% CI – 19.1 – 30.9 months), while the PFS 2 in patients progressed at other sites was 20.0 months (95% CI – 18.3 – 23.3 months) (*p* = 0.38). The OS was 27.2 months (95% CI – 24.9 – 31.3 months) in patients with CNS progression compared to 22.2 months (95% CI – 19.0 – 30.1 months) in the patients progressed other sites (*p* – 0.57). The survival curves are provided in Supplementary Data Figures 3 and 4.

#### Outcomes of patients with other resistance patterns

Patients with SCLC transformation received platinum plus etoposide combination along with continuation of TKI and all of them initially responded but later had a progression with a median OS from the point of transformation being 14.2 months. In patients who had new sensitising mutations, the continuation of gefitinib (with one patient being started on osimertinib in view of progression in brain) and chemotherapy added if rapid response was needed, yielded an addition PFS2 of nearly 8 months in this group. One patient having ALK mutation was started on crizotinib and it has given a progression-free interval of 3.3 years in that patient.

## Discussion

In our study, in patients with *EGFR* mutant NSCLC treated with either gefitinib or gefitinib plus chemotherapy in the first line setting, the most common pattern of resistance was T790M mutation noted in 30% patients. In the literature, prior studies also have shown the predominant pattern of resistance as T790M in exon 20, being 49% in the study by Sequist *et al* [22] 62% in the study by Yu *et al* [23] and 58% in the IMPRESS trial [25]. The emergence of T790M in the gefitinib alone arm (37%) was relatively similar to these studies, which also used TKI alone. The emergence of T790M as a pattern of resistance was significantly more in the gefitinib alone arm (37%) than the gefitinib plus chemotherapy arm (19%). This phenomenon of suppression of the emergence of the T790M clone in the arm receiving chemotherapy has not been previously documented.

Among the studies that compared TKI to TKI plus chemotherapy, NEJ009 (gefitinib plus chemotherapy versus gefitinib alone) did not report the resistance patterns, in IMPRESS trial (gefitinib plus chemotherapy versus placebo plus chemotherapy), the T790M was noted in 62% of the patients without any difference between the two arms. The comparison was also studied in the preliminary data published in the FLAURA 2 trial (osimertinib versus osimertinib plus chemotherapy) [28]. The patterns noted in the FLAURA 2 were not significantly difference between the two arms, although numerically higher EGFR C797S was observed in the osimertinib monotherapy arm. The resistance patterns observed were mesenchymal epithelial transition factor (MET) amplification (11.9%), EGFR C797S (8.7%), uncommon EGFR mutations (2.3%), Phosphatidylinositol – 4,5 – bisphosphate 3-kinase catalytic subunit alpha (PIK3CA) (6.3%), CDK 4/6 amplification (5.5%), Cyclin D/E amplification (3.2%), human epidermal growth factor receptor 2 (HER2) amplification (3.2%), v-raf murine sarcoma viral oncogene homolog B (BRAF) V600E (3.2%), rearranged during transfection fusion (3.2%), BRAF fusion (3.2%), ALK fusion (1.6%), other fusions (5.5%) and no known resistance pattern noted in 42% patients [28]. These patterns were different from our study as osimertinib is active against the T790M and it was, therefore, not noted in the FLAURA 2 study. The other changes being less frequently noted might be due to the less frequent use of NGS in our study.

The lower rates of T790M observed in the present study, might be due to the fact that patients in one arm received chemotherapy along with TKI and being a biologically different population. Among the patients with T790M and those with CNS progression in whom the *EGFR* sensitising mutation was retained, 62 patients received osimertinib in the 2nd line and they had a PFS of 23.3 months compared to 18.9 months in the rest of the cohort (*p* – 0.28). These data were similar to the APPLE study, where osimertinib was used sequentially after emergence of T790M in circulating tumour DNA on gefitinib (22.1 months in osimertinib upfront versus 21.9 months in the sequential arm) [26]. This provides the rationale for sequential therapy, especially in resource constrained settings.

Among the other mechanisms of resistance noted in our study was loss of prior sensitising mutation, seen in 21%, which was noted in 27% in a smaller study done by Tabara *et al* [27]. It was also noted in a case report [28]. This loss of prior sensitising mutations was higher in the chemotherapy plus gefitinib arm (32%) compared to the gefitinib alone arm (17%) (*p* – 0.015). This pattern of loss of sensitising mutation more in the TKI plus chemotherapy treated patients has not been previously documented. This might be due to the possible conversion to a non-molecularly driven tumour implications for subsequent lines of therapy.

Histological transformation was seen in 6% (12 patients), of which nine had a transformation from adenocarcinoma to SCLC. Overall, in the literature transformation has been reported to be between 3% and 10% [22, 23]. The rate of SCLC transformation was not significantly different between the two treatment groups. Patients with SCLC transformation received platinum plus etoposide combination along with continuation of TKI and all of them initially responded but later had a progression with a median OS from the point of transformation being 14.2 months. It has been noted as 10.9 months in the study by Marcoux *et al* [29]. Patients with transformed SCLC in the setting of *EGFR* mutant NSCLC appear to have relatively good prognosis as compared to de novo SCLC.

New sensitising mutations were noted in 3% cases. This number was similar to the previous studies [22, 23]. This number was noted to be 4% in the FLAURA 2 trial [30]. In our study, these patients were continued on gefitinib (with one patient being started on osimertinib in view of progression in brain) and chemotherapy added if rapid response was needed. This has given an additional PFS2 of nearly 8 months.

Molecular mutations other than EGFR were noted but the baseline NGS was not performed. But these alterations were known mechanisms of resistance. Tumour protein 53 (TP53) mutation was found in 2% of the cases. The patients having TP53 mutations were known to increased histological transformation rate and early progression and acquired resistance of EGFR based therapy [31]. In our study, we noted 2 cases of SCLC transformation having TP53 mutations. The other changes include – PIK3CA mutation, ALK mutation, cyclin-dependent kinase inhibitor 2A (CDKN2A) mutation and ROS mutation being noted in 1% each. PIK3CA mutation was noted in 5% cases in the study by Sequist *et al* [22] and 6% in the FLAURA 2 study. ALK mutation was noted after progression on second-line osimertinib; in this case, the addition of crizotinib to osimertinib led to stabilisation of disease [32]. In our study, also crizotinib addition was also done in one patient and has shown an excellent progression free interval of about 3.3 years. CDKN2A was noted in one of the studies as a resistance mechanism post 2nd line osimertinib, but alterations in the wider group of cell cycle regulators were noted in 10%–12% cases [33, 34]. ROS fusion was noted previously in one case report where addition of crizotinib has led to a partial response [35]. The other mechanisms noted in previous studies which we did not find in our cohort were - MET amplification in about 3%–5% cases, HER2 amplification in about 3% cases, PIK3CA mutation in about 1%–5% cases and Beta catenin amplification in 2%–5% cases.

No new pattern of resistance was noted in 36% of the cases. This was in line with what was seen in the previous studies, i.e., 30%–40% [22, 23]. 30% in the study by Yu *et al* [23] and 40% in the study by Sequist *et al* [22] Modern analysis like NGS were not done in all the available patients in these studies due to lack of samples or feasibility, which may be the reason for not identifying molecular cause of resistance.

The limitations of our study include the lack of use of NGS which was expensive and not affordable by majority of patients at the study period might have led to missing the patterns of resistance in some patients. About 22% of patients were not included in the final analysis as the repeat biopsy was not feasible. Some patients in this group had early death post progression and the analysis in this group might have helped to note the molecular changes that led poor outcomes.

## Conclusion

Our study describes the patterns of resistance in *EGFR* mutant NSCLC and will help guide the future therapeutic options. Patients with SCLC transformation, T790M mutation, ALK mutation and ROS fusion had meaningful outcomes with molecular therapies post progression. Our study emphasises the need to perform repeat biopsy and molecular analysis at PD.

## List of abbreviations

ALK IHC, Anaplastic lymphoma kinase – immunohistochemistry; *BRAF* gene, v-raf murine sarcoma viral oncogene homolog B; *CDKN2A* gene, Cyclin-dependent kinase inhibitor 2A; cfDNA, Circulating free deoxyribonucleic acid; CNS, Central nervous system; *EGFR*, Epithelial growth factor receptor gene; HER2, Human epidermal growth factor receptor 2; NGS, Next generation sequencing; NSCLC, Non-small cell lung cancer; OS, Overall survival; PD, Progressive disease; PFS, Progression free survival; PFS 2, Progression free survival 2; *PIK3CA* gene, Phosphatidylinositol – 4,5 – bisphosphate 3- kinase catalytic subunit alpha; RT-PCR, Reverse transcription polymerase chain reaction; SCLC, Small cell lung carcinoma; TKIs, Tyrosine kinase inhibitors; TMC, Tata Memorial Center; TP53, Tumour protein 53.

## Conflicts of interest

None declared.

## Funding

No external funding was received for this study.

## Figures and Tables

**Table 1.** Baseline demographic and molecular characteristics.

Patient characteristics	Number of patients (%) (*N* – 350)
Age, years Median (Interquartile range)	54.5 (47.0–60.1)
Sex Female Male	169 (48.3) 181 (51.7)
Smoking history Never smoker Former smoker Current smoker	295 (84.3) 40 (11.4) 15 (4.3)
Adequacy of baseline biopsy Adequate Inadequate	324 (92.6) 26 (7.4)
Histopathology Adenocarcinoma Squamous Adeno squamous Sarcomatoid carcinoma	338 (96.6) 4 (1.1) 7 (2.0) 1 (0.3)
Molecular test done at baseline EGFR RT PCR^#^ only EGFR RT PCR + ALK IHC ^^^	99 (28.3) 251 (71.7)
**Types of EGFR mutation at baseline**	**Frequency**
**Classical mutation – 335 (95.7)**	
Exon 19 in-frame deletion	216 (61.7)
Exon 21 L858R mutation	119 (34)
**Rare mutations – 5 (1.5)**	
Exon 18 (G 719 X)	3 (0.9)
Exon 21 L861 Q	1 (0.3)
Exon 20 (T 790 M)	1 (0.3)
Compound mutation	10 (2.8)*

# EGFR RT PCR – epidermal growth factor receptor real time reverse transcriptase polymerase chain reaction

^ ALK IHC – anaplastic lymphoma kinase receptor immunohistochemistry

* exon 20 (T790M) and exon 21 L858R mutation – 4 (1.2%); exon 20 (T790M) and exon 18 (G719X) – 1 (0.3%); exon 19 (del) and exon 21 (L858R) positive – 1 (0.3%); exon 19 deletion and exon 21 L861Q – 1 (0.3%); exon 18 (G719X) and exon 21 (L861Q) – 1 (0.3%); exon 20 S768I and exon 21 L858R mutation – 1 (0.3%); exon 20 (S768I) and exon 18 (G719X) mutation – 1 (0.3%); exon 21 L858R and exon 21 L861Q – 1 (0.3%); exon 20 (T790M) and exon 19 (del)

**Table 2.** Comparison of resistance pattern between the treatment groups.

Pattern of resistance	Gefitinib alone arm	Gefitinib + chemotherapy arm	*p* value
**T790M -**	**44 (37%)**	**17 (19%)**	**(*p* – 0.008)**
T790M in Exon 19 deletion	31	9	
T790M in Exon 21 L858R mutation	11	8	
T790M and Exon 21 L858R mutation in Exon 19 deletion	1	0	
T790M	1	0	
**New sensitizing mutation**	**6 (5%)**	**1 (1.1%)**	**(*p* – 0.12)**
New Exon 19 deletion in Exon 21 L858R mutation	4	1	
New Exon 21 L861Q in Exon 21 L858R mutation	1	0	
New Exon 20 S768I mutation in Exon 21 L858R mutation	1	0	
**Loss of prior sensitizing mutation**	**21 (17.6%)**	**28 (32%)**	**(*p* – 0.015)**
Loss of Exon 19 deletion	15	19	
Loss of Exon 21 L858R mutation	5	9	
Loss of Exon 18 G719X loss in G719X in Exon 18 G719X and Exon 21 L858R mutation	1	0	
**Histological transformation**	**5 (4.2%)**	**7 (8.1%)**	**(*p* – 0.12)**
Adenocarcinoma to squamous cell carcinoma	1	0	
Adenocarcinoma to small cell carcinoma	3	6	
Adenocarcinoma to neuroendocrine carcinoma	0	1	
Squamous cell carcinoma to adenocarcinoma	1	0	

*Factors affecting the outcome in the patients progressed on EGFR-based therapy*

**Table 3.** Details of results of repeat biopsy in patients who had PD.

Type of biopsy	Number of patients (%) *N* - 206
Only tissue biopsy	35 (17)
Both tissue and liquid biopsy	120 (57.4)
Only liquid biopsy	37 (18)
Only cell block	6 (2.9)
Both cell block and liquid biopsy	8 (3.9)
**Reason for inability to obtain a biopsy**	**Number of patients (*n* = 63)**
Biopsy not feasible (patient refusal/inaccessible site)	24 (38.2)
Oligoprogression and received radical therapy	7 (11.1)
Defaulted	4 (6.3)
Lost to follow-up	9 (14.3)
Poor general condition	19 (30.1)
**Histopathology at progression**	**Number of patients (*n* = 171)**
Adenocarcinoma	135 (79)
Squamous cell carcinoma	2 (1.2)
Small cell carcinoma	7 (4.1)
Mixed adenocarcinoma + small cell carcinoma	2 (1.2)
Adenosquamous	3 (1.8)
Adenocarcinoma with neuroendocrine differentiation	1 (0.6)
Necrotic tissue^*^	4 (2.3)
Non malignant^#^	15 (8.7)
**Patients with histological transformation**	**Number of patients (*n* = 12)**
Adenocarcinoma to squamous cell carcinoma	1 (8.3)
Adenocarcinoma to small cell carcinoma	9 (75)
Adenocarcinoma to neuroendocrine carcinoma	1 (8.3)
Squamous cell carcinoma to adenocarcinoma	1 (8.3)
**Type of molecular testing done on re-biopsy sample**	**Number of patients (%) *N* – 206**
• NGS done NGS alone RT-PCR + NGS RT-PCR + ALK IHC + NGS	22 (10.7) 2 (1) 18 (8.7) 2 (1)
• NGS not done RT-PCR + ALK IHC RT-PCR alone	184 (89.3) 5 (2.4) 179 (86.9)

* These patients having necrotic tissue on biopsy found to have circulating tumour cells and were taken into analysis based on the results of circulating free deoxyribonucleic acid (cfDNA) analysis

# 15 of the 17 patients with no evidence of malignancy noted on repeat tissue biopsy were taken into analysis based on the results of cfDNA analysis.

## Supplementary data

As per May 2024, the updated data shows the following outcomes – as charted in Table S1.

**Table S1.** Outcomes.

Outcome		No.	%
No PD		9	2.5
LFU		5	1.43
PD		11	3.14
Died		325	92.8

### PFA analysis

Using Kaplan meir analysis and log rank test analysis to calculate the hazard ratio using progression, death or date of last follow-up as the event with the event occurring first being taken. The PFS for the entire cohort is 15.3 months (95% CI, 13.9 to 16.7 months). The addition of pemetrexed and carboplatin to geftinib improved the PFS from 12 months (95% CI, 10.9 to 13.4 months) to 19 months (95% CI, 16.4 to 21.4 months) with HR for death or progression being 0.52 (95% CI, 0.39 to 0.66; p < 0.001). The differences seen by the Kaplan meir analysis were noted to be significant by the, log rank test. The PFS among various groups was charted as follows in the Table S2.

**Table S2.** PFS among various groups.

	Number of patients (%) *N = 350*	Overall cohort	Gefitinib group	Gefitinib + chemotherapy group
Exon 19 indel	216	16.68 months	12.8 months	21.18 months
Exon 21 L858R	102	13.6 months	11.4 months	15.8 months
Exon 21 (L858R/L861Q)	16	12.5 months	12.2 months	13.5 months
Exon 20 T790M + Exon 21 L858R	3	10.6 months	9 months	11.5 months
Exon 20 T790M – upfront	6	9.4 months	8.9 months	10.3 months

### Site of progression

The radiological evaluation was done in all the patients suspected of clinical progression and in some patients as part of regular monitoring. In three patients, cerebro-spinal fluid (CSF) analysis revealed disease progression. The sites of progression were noted as follows in the evaluation – in Table S3.

**Table S3.** Sites of progression.

Site of PD	Patients with PD
Primary	54 (20.3%)
Primary and metastatic	69 (26%)
At metastatic site	26 (9.8%)
New lesions	44 (16.6%)
New or increased pleural or pericardial fluid	21 (7.9%)
CSF	3 (1.13%)
Brain	48 (18.1%)

### Site of biopsy

Based on the site of progression and feasibility of biopsy, biopsy or cell block was obtained. The sites of biopsy are charted in Table S4.

**Table S4.** Site of biopsy.

Site of biopsy	Number of patients
Lung primary	115
Pleural fluid cell block	15
CSF cell block	4
Lymph node	4
Liver	18
Pleura	6
Bone	5
Ascitic fluid cell block	3
Brain tissue	1
Total	171

### Complications of biopsy

Out of the 115 patients who had lung biopsy, 7 had minimal pneumothorax, 6 needed interventions due to pneumothorax, 1 patient had dizziness and bradycardia and 1 had severe post procedural pain. Of the others only one patient of liver biopsy had significant post procedural pain. The details are tabulated in Table S5.

**Table S5.** Complications of biopsy.

Complication – all at lung biopsy	No of patients
Minimal pneumothorax	7
Pain	2
Pneumothorax needing intervention	6
Dizziness with Bradycardia during procedure	1

### Molecular analysis of the 206 patients available for analysis

In the molecular analysis, T790M was noted as the predominant molecular alteration noted accounting for 61 patients being 29.6% of the cases with resistance Table S6.

**Table S6.** Molecular analysis of the 116 patients with new molecular alterations.

Type of molecular alteration	Number of patients (*n* = 116)
New mutation of T790M in patient with Exon 19 deletion	40
Loss of exon 19 deletion in patient with Exon 19 deletion	34
Loss of L858R in patient of L858R mutation	14
New mutation of T790M in patient with Exon 21 L858R mutation	19
New Exon 19 deletion in patient with L858R mutation	5
Loss of G719X in patient with G719X mutation	2
New mutation of L858R with T790M in patient with exon 19 deletion	1
New mutation of L861Q in case of L858R mutation	1
Total	116

In rest of the patients- N -90

**Table S7.** Molecular analysis of the 90 patients with retained molecular alterations.

Type of molecular alteration	Number of patients (*n* = 90)
Exon 19 deletion retained	55
Exon 21 L858R retained	31
Exon 21 L861Q retained	1
S768I retained	1
G719X rtained	1
T790M and exon 21 L858R retained	1
Total	90

Additional mutation noted on NGS in 10 patients out of 17 in which NGS was performed Table S8.

**Table S8.** Additional molecular analysis noted on NGS.

Mutation	Number of patient	Type of molecular EGFR mutation noted
TP53 mutation	7	5 had Exon 19 deletion retained , 2 had loss of exon 19 deletion
CDKN2A deletion	1	Retained exon19 deletion
PIK3CA	1	Loss of L858R
ROS FUSION	1	Loss of exon 19 deletion
ALK mutation	1	Loss of exon 19 deletion

### Overlapping resistance patterns noted

1. 1 case of SCLC with loss of exon 19 deletion
2. 1 case of SCLC with new mutation of S768I with prior L858R retained
3. 2 cases of SCLC with TP53 mutations
4. 1 case of Neuroendocrine carcinoma transformation with T790M muatation
5. 1 case of Adenocarcinoma to squamous cell carcinoma transformation along with T790M mutation
6. 1 case of loss of exon 19 deletion with ALK mutation
7. 1 case of loss of exon 19 deletion with ROS fusion
8. 1 case of loss of L858R mutation with PIK3CA amplification
9. 2 cases of loss of exon 19 deletion with gain of TP53 mutation
